# Supplementary material for: An integrase toolbox to record gene-expression during plant development
Source: Nat Commun. 2023 Apr 3;14:1844. doi: 10.1038/s41467-023-37607-5 (PMC10070421; doi:10.1038/s41467-023-37607-5)
Supplement: Supplementary file 1 — Supplementary Information [file 41467_2023_37607_MOESM1_ESM.pdf]

An integrase toolbox to record gene-expression during plant development

Guiziou *et al.*

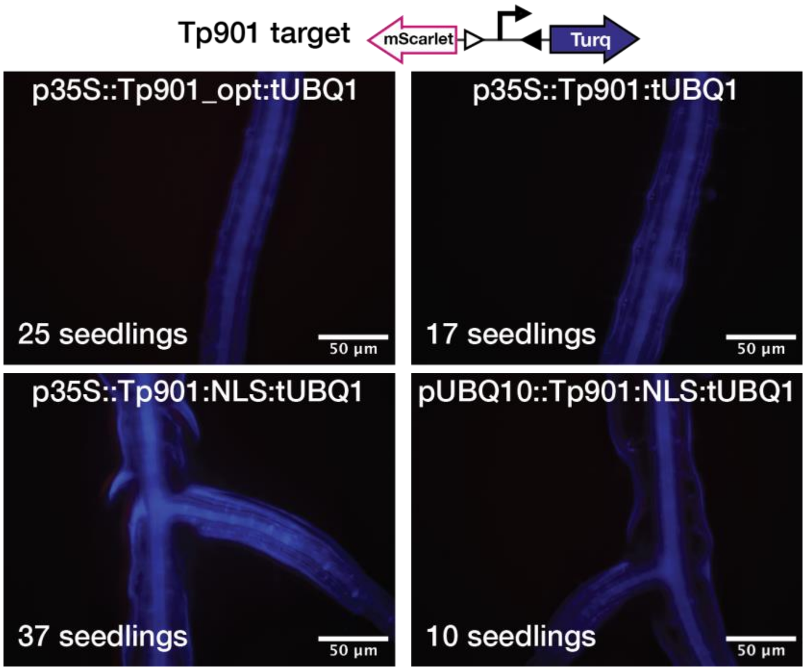

Supplementary Figure 1. Tp901 integrase does not work well in *Arabidopsis*. Tp901 target line was transformed with Tp901 integrase constructs. If the integrase was active the target should swtch from mTurq to mScarlet expression. No mScarlet was ever detected. T1 expression patterns were analyzed by microscopy. Each image corresponds to a representative image for each integrase construct, the integrase construct is mentioned on the top and the number of seedling characterization on the bottom left. The microscopy image is an overlay of the blue and red fluorescence channel. The number of seedlings indicated corresponds to the number of seedlings characterized.

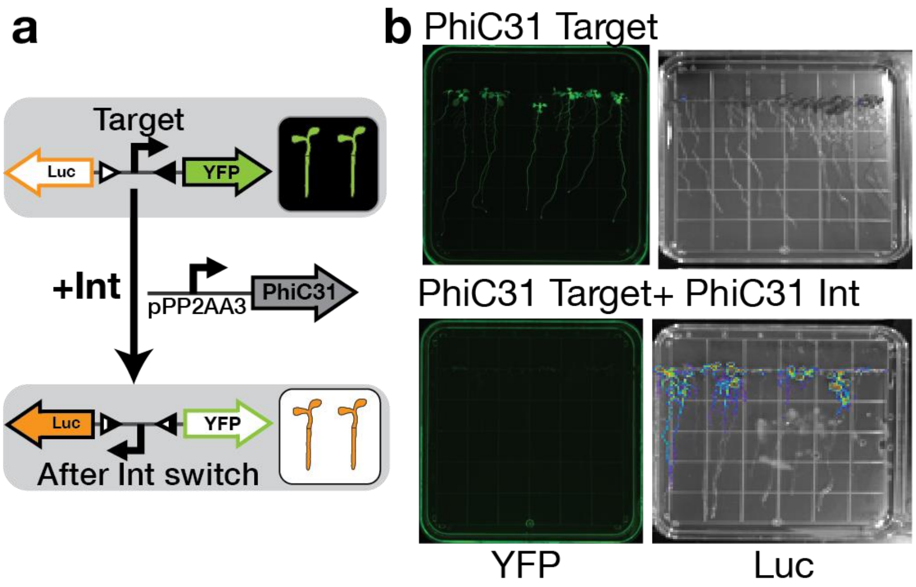

Supplementary Figure 2. Integrase target for macroscopic analysis. (a) The PhiC31 target [1] switches from YFP to Luciferase expression, allowing to image the full seedling under gel imager for YFP or night owl after luciferin treatment. We used pPP2AA3 to drive PhiC31 integrase expression constitutively. (b) Images of T2 seedling under an Azure c600 Gel imaging system (for YFP fluorescence) (left) and NightOWL LB 983 *in vivo* imaging system (for Luciferase) after luciferin treatment (right), on the top are seedlings with target and no integrase at the bottom seedlings with target and integrase.

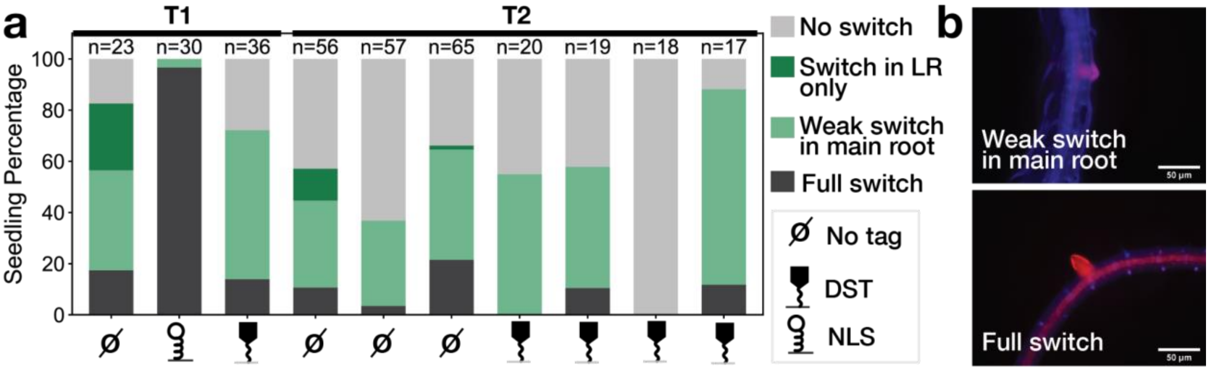

Supplementary Figure 3. Characterization of pLBD16 integrase switch constructs. (a) Phenotyping of T1 seedlings with constructs in PhiC31 target line, constructs are PhiC31 with pLBD16, and various tuning tags (label at the bottom of the graph): no tag, DST, or NLS (legend on the right). The graph corresponds to the percentage of seedlings in each of the defined phenotype categories, such as no switch corresponding to no mScarlet expression in the root, switch in LR only: mScarlet expression only in the lateral root, weak switch in the main root: mScarlet expression in few cells in the main root (corresponding to the image in b), full switch: mScarlet expression everywhere in the root (corresponding to the image in b). The number of seedlings characterized for each construct is mentioned at the top of the bar in the graph. (b) Representative image of the seedling in the weak switch in main root and full switch phenotypic categories. Microscopy images are an overlay of the blue and red channels. Source data are provided as a Source Data file.

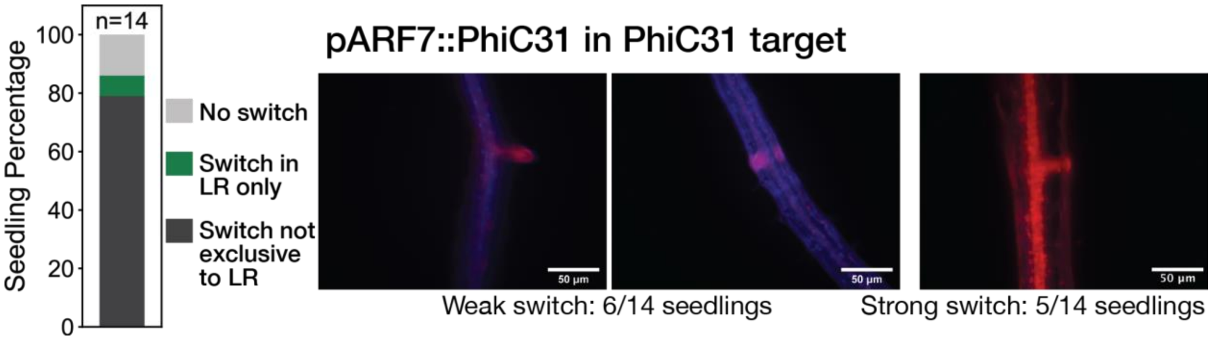

Supplementary Figure 4. Characterization of pARF7::PhiC31 switch. 14 T1 seedlings of pARF7::PhiC31 in PhiC31 target line were characterized. On the left, the bar graph corresponds to the percentage of seedlings in each of the phenotypic categories, no switch in light gray, switch only in LR in green, and switch not exclusive to LR in dark gray. On the right are representative images of seedlings with switch not exclusive to LR, either a weak switch (6/14 seedlings) or a strong switch everywhere (5/14 seedlings). Source data are provided as a Source Data file.

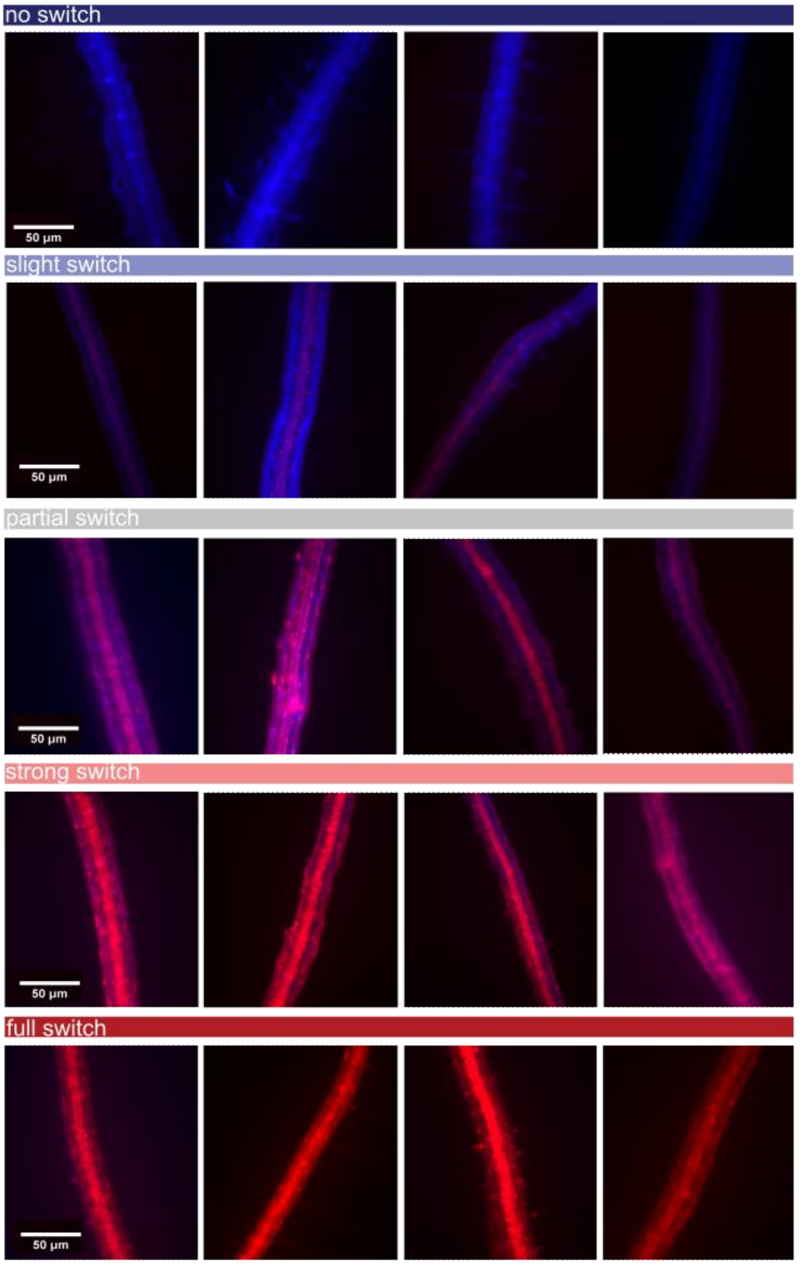

Supplementary Figure 5. Example images of switching categories for tuning data. For evaluating the tuning results we sorted each seedling into one of five categories (no switch, slight switch, partial switch, strong switch, full switch). Within each category variation is present. The 20 seedlings pictured above represent the spectrum of constitutive switching levels we observed in characterizing integrase tuning parts. The scale bar is shown in the left-most image in each row and the scale applies through the whole row.

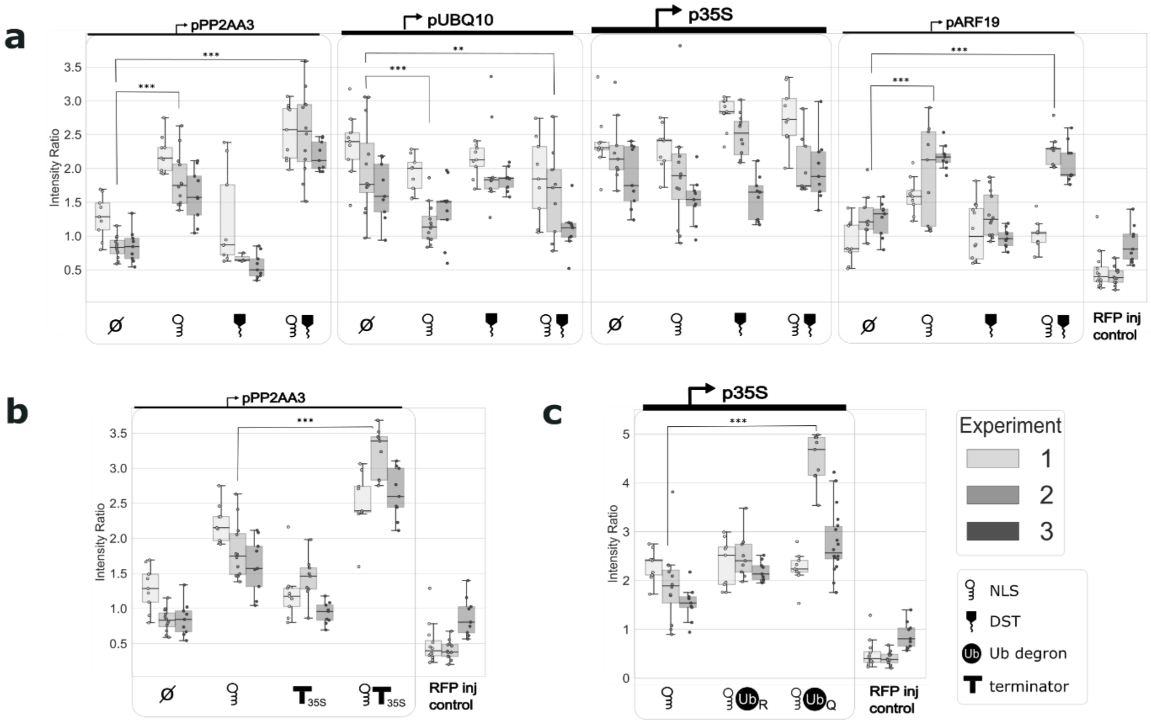

Supplementary Figure 6. The effect of tuning modifications differ between *N. benthamiana* and *Arabidopsis*: (**a-c**) Tuning was tested in *N. benthamiana* through tobacco injection. The integrase target switches from a Luc reporter to YFP and an RFP injection control was co-injected with each construct. The metric for level of switching is the ratio of YFP to RFP. Each tuning construct was injected into 3 leaves per experiment. Three punches were taken from each leaf and the resultant fluorescence was measured with a plate reader. Each point on the boxplot represents one leaf punch. Each box represents one of three replicate experiments performed for each construct. The box represents the middle two quartiles of the data and the whiskers represent the lowest and highest quartiles. The horizontal line within the box represents the median. The minimum and maximum values are shown by the termination of the whiskers, except in the case of some outliers which are shown as individual points. Tuning parts tested were (**a**) NLS and DST (*p* values left to right are 0, 0, 0.0009, 0.0097, 0, and 0). , (**b**) varied terminator (*p* value = 0), and (**c**) Ub degron (*p* value = 0). The data were tested for significance using a two-sidedn ANOVA and post-hoc Tukey's HSD test (*: $p<0.05$, **: $p<0.01$, ***: $p<0.001$). Source data are provided as a Source Data file.

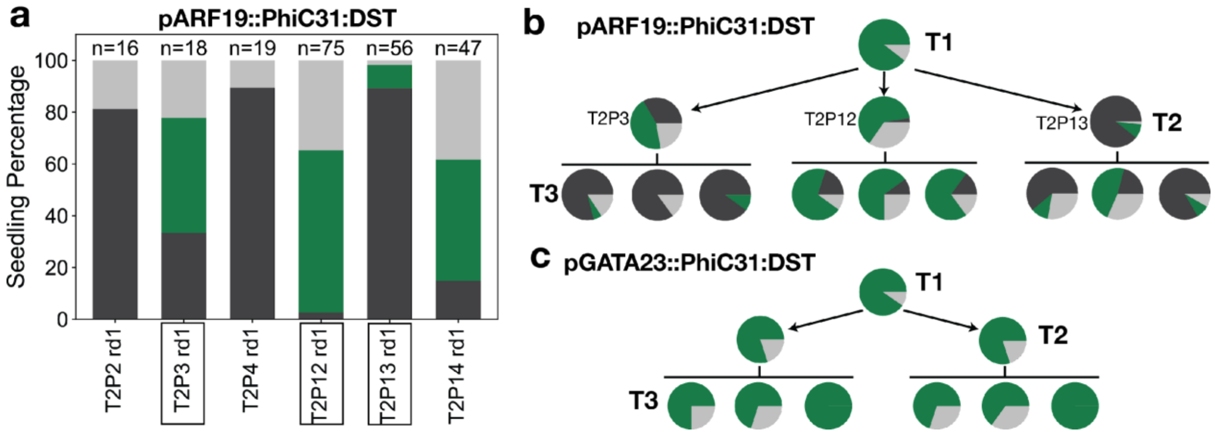

Supplementary Figure 7. Additional data for pARF19::PhiC31:DST and pGATA23::PhiC31:DST. (a) Phenotyping of T2 seedlings from T1 lines with pARF19::PhiC31:DST in PhiC31 target line. The graph corresponds to the percentage of seedlings in each of the defined phenotype categories, such as no switch corresponding to no mScarlet expression in the root, switch in LR only: mScarlet expression only in the lateral root, switch not exclusive to LR: mScarlet expression in the main root. The number of seedlings characterized for each construct is mentioned at the top of the bar in the graph. The lines used in Figure 4 have their name boxed. (b) and (c) Phenotype of T1, T2, T3 plants from pARF19::PhiC31:DST (b) and pGATA23::PhiC31:DST (c) constructs in PhiC31 target line. The pie charts are another representation of the previous phenotype bar graph representing the percentage of seedlings in each of the defined phenotype categories. From each generation, three seedlings with the LR-only switch phenotype were kept to generate the next generation. Source data are provided as a Source Data file.

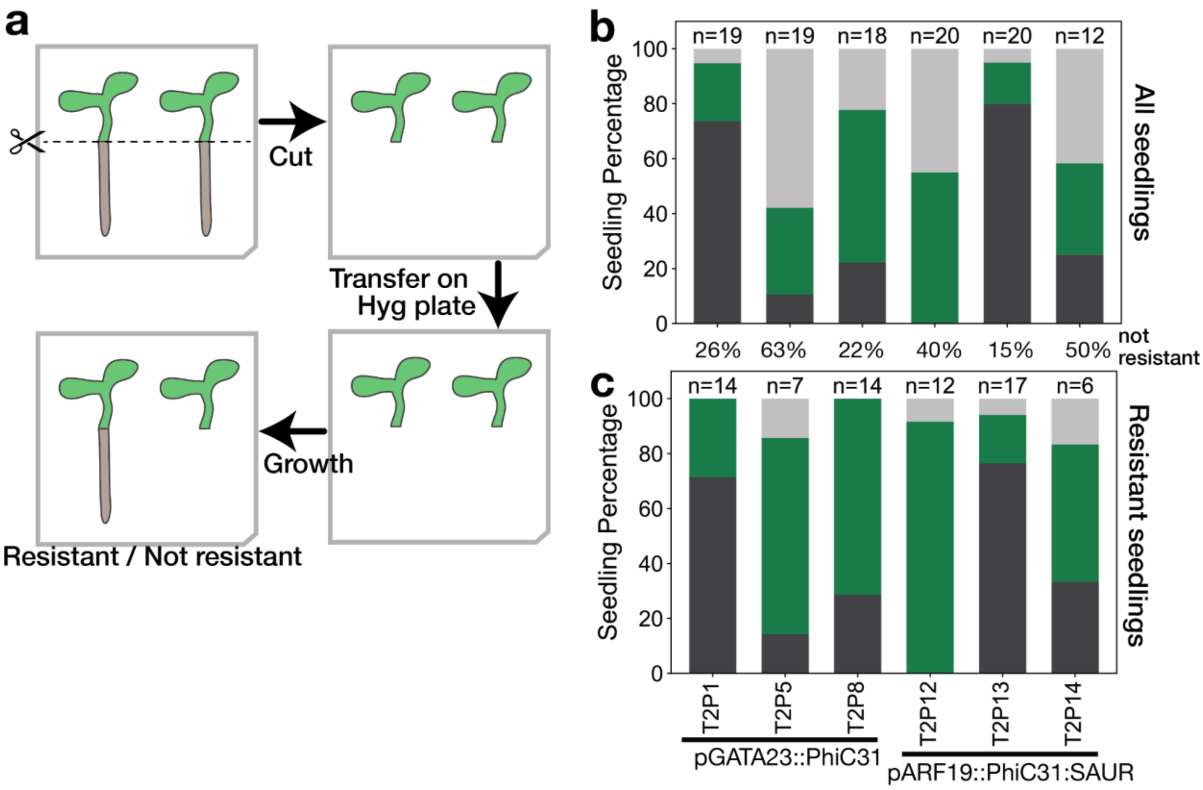

Supplementary Figure 8. Post-phenotyping selection of seedlings. (a) Process to determine post-characterization if a seedling is resistant to hygromycin. Post microscope characterization, seedlings were cut between the hypocotyl and the root. The aerial tissue (hypocotyl and cotyledons) were transferred to a hygromycin plate and grown for seven days. Resistant seedlings grew new roots. (b) and (c) Phenotype of T2 seedlings for pGATA23::Phic31 and pARF19::PhiC31:SAUR constructs in PhiC31 target line. Labels of the lines are at the bottom, T2PX corresponds to the name of the specific line. (b) is the phenotype characterization of one round of T2 seedlings for those lines (number at the top of the bars). (c) is the same data from b with only Hyg resistant seedlings included. The percentage of seedlings which are not resistant is represented between both graphs. The graph corresponds to the percentage of seedlings in each of the defined phenotype categories: no switch (light gray); LR only (green); not exclusive (dark gray). Source data are provided as a Source Data file.

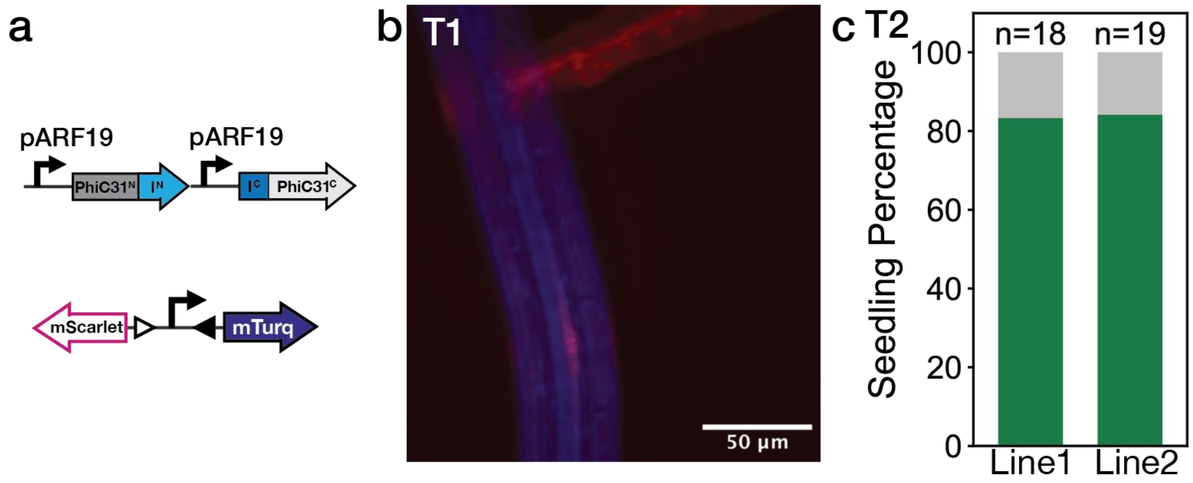

Supplementary Figure 9. Split-intein system with ARF19 promoter. (a) Schematic of the ARF19 promoter driving the two split-intein parts and the PhiC31 integrase target composing this Arabidopsis line. **(b)** 10 T1 seedlings were imaged, here a representative image of a seedling with switch observed in a fully emerged lateral root and in an early stage of LR development. **(c)** Phenotyping of T2 seedlings from two T1 lines with pARF19 driving the two split-intein parts in PhiC31 target line. The graph corresponds to the percentage of seedlings in each of the defined phenotype categories, such as no switch corresponding to no mScarlet expression in the root (in gray), switch in LR only: mScarlet expression only in the lateral root (in green), switch not exclusive to LR: mScarlet expression in the main root (in black - none here). The number of seedlings characterized for each construct is mentioned at the top of the bar in the graph. Source data are provided as a Source Data file.

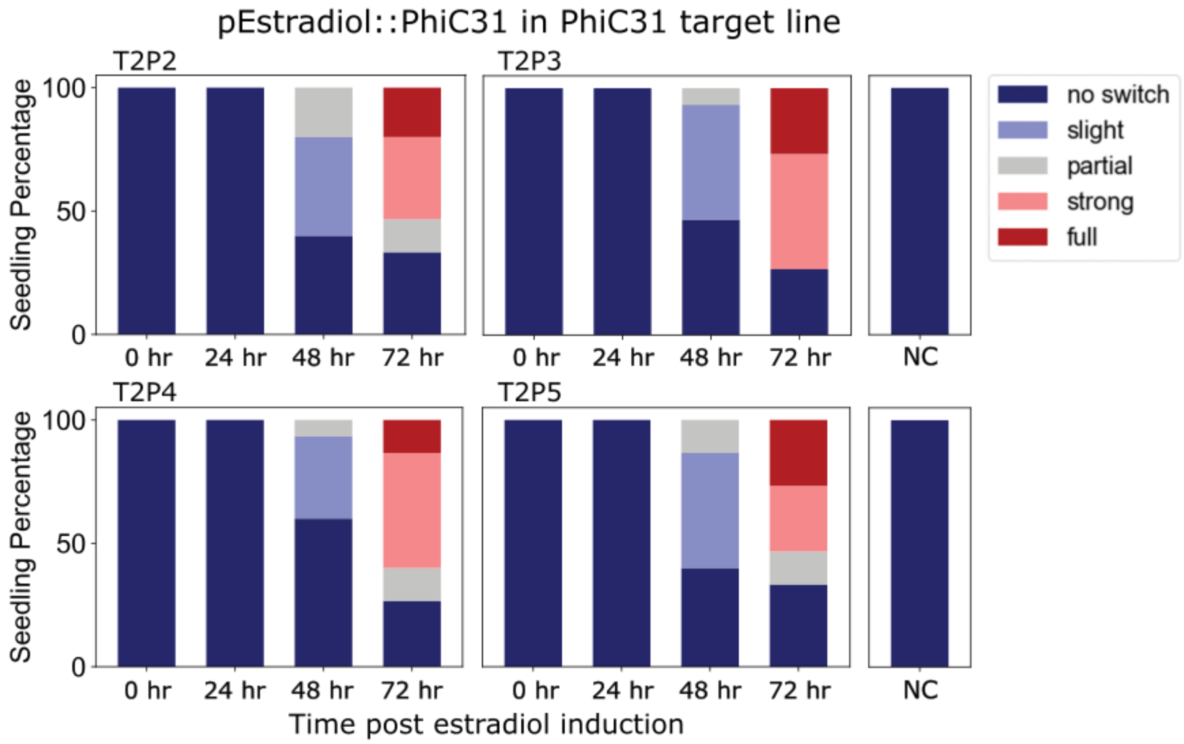

Supplementary Figure 10. Estradiol induction of PhiC31 integrase in T2 seedlings. T2 seeds were collected from T1 plants which were not treated with estradiol. Four T2 lines were tested: T2P2, T2P3, T2P4, and T2P5 with 15 seedlings screened for each line. Seedlings were imaged and classified based on switching level as per Supplementary Figure 5 at 0hr, 24hr, 48hr, and 72hr post-estradiol induction. Source data are provided as a Source Data file.

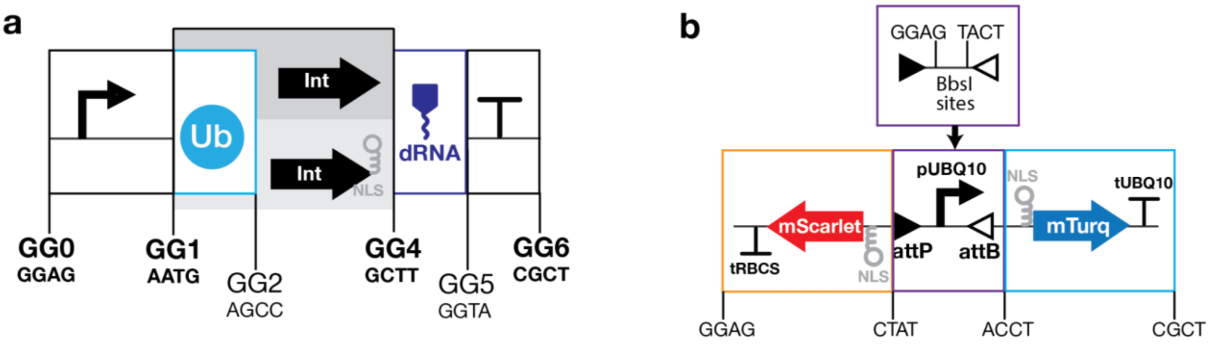

Supplementary Figure 11. Cloning strategy based on golden gate assembly. (a) Cloning strategy for the integrase construct with the BsaI spacer between each part specified. (b) Cloning strategy for the integrase construct. The central part with the integrase sites and promoter is constructed by golden gate assembly with BbsI enzyme to add the promoter to the synthetic fragment with the integrase sites only.

Supplementary reference

1. Bernabé-Orts, J. M. *et al.* A memory switch for plant synthetic biology based on the phage ϕC31 integration system. *Nucleic Acids Res.* **48**, 3379–3394 (2020).
