# Supplementary material for: An integrase toolbox to record gene-expression during plant development
Source: Nat Commun. 2023 Apr 3;14:1844. doi: 10.1038/s41467-023-37607-5 (PMC10070421; doi:10.1038/s41467-023-37607-5)
Supplement: Supplementary file 3 — Description of Additional Supplementary Files [file 41467_2023_37607_MOESM3_ESM.pdf]

Description of Additional Supplementary Files

File Name: Supplementary Data 1
Description: List of primers with primer number, primer name, primer sequence and the purpose of the primer.

File Name: Supplementary Data 2
Description: Construct list with sequences. For level0, the golden gate linkers, the primers used to amplify the sequences, and the origin of the sequence are provided. For level1, the level0 used to generate the level1 are specified, as well as the agro number.
