# Supplementary material for: An integrase toolbox to record gene-expression during plant development
Source: Nat Commun. 2023 Apr 3;14:1844. doi: 10.1038/s41467-023-37607-5 (PMC10070421; doi:10.1038/s41467-023-37607-5)
Supplement: Supplementary file 6 — Reporting Summary [file 41467_2023_37607_MOESM6_ESM.pdf]

nature portfolio

Corresponding author(s): Jennifer Nemhauser

Last updated by author(s): 2/10/2023

Reporting Summary

Nature Portfolio wishes to improve the reproducibility of the work that we publish. This form provides structure for consistency and transparency in reporting. For further information on Nature Portfolio policies, see our Editorial Policies and the Editorial Policy Checklist.

Statistics

For all statistical analyses, confirm that the following items are present in the figure legend, table legend, main text, or Methods section.

n/a	Confirmed	
☐	☒	The exact sample size (*n*) for each experimental group/condition, given as a discrete number and unit of measurement
☐	☒	A statement on whether measurements were taken from distinct samples or whether the same sample was measured repeatedly
☐	☒	The statistical test(s) used AND whether they are one- or two-sided<br>*Only common tests should be described solely by name; describe more complex techniques in the Methods section.*
☒	☐	A description of all covariates tested
☐	☒	A description of any assumptions or corrections, such as tests of normality and adjustment for multiple comparisons
☐	☒	A full description of the statistical parameters including central tendency (e.g. means) or other basic estimates (e.g. regression coefficient) AND variation (e.g. standard deviation) or associated estimates of uncertainty (e.g. confidence intervals)
☐	☒	For null hypothesis testing, the test statistic (e.g. *F*, *t*, *r*) with confidence intervals, effect sizes, degrees of freedom and *P* value noted<br>*Give P values as exact values whenever suitable.*
☒	☐	For Bayesian analysis, information on the choice of priors and Markov chain Monte Carlo settings
☒	☐	For hierarchical and complex designs, identification of the appropriate level for tests and full reporting of outcomes
☒	☐	Estimates of effect sizes (e.g. Cohen's *d*, Pearson's *r*), indicating how they were calculated

Our web collection on statistics for biologists contains articles on many of the points above.

Software and code

Policy information about availability of computer code

Data collection	Imaging software: NIS-Elements 5.30.03 (Build 1549) for confocal microscopy and LAS AF version 2.6.0.7266.2 for fluorescent microscopy. Plate reader software: Sparkcontrol v2.3 from TECAN. Software for Luciferase imaging: indiGO version 2.0.5.0. Software for YFP imaging in all plants: AzureTM cSeries version 1.9.8.0403
Data analysis	To analyze the data in the study, two Python scripts were used. Python data analysis script which includes statistical tests and plotting functions was run in version 3.9.1 and with the following package dependencies: pandas (version 1.5.3), scipy.stats (version 1.10.0), matplotlib.pyplot (version 3.6.3), matplotlib.colors (version 3.6.3), scikit_posthocs (version 0.21), and numpy (version 1.24.2). One ImageJ macro was used for image analysis (ImageJ version 1.53c). Python and ImageJ script are available on GitHub [http://doi.org/10.5281 zenodo.7612666]

For manuscripts utilizing custom algorithms or software that are central to the research but not yet described in published literature, software must be made available to editors and reviewers. We strongly encourage code deposition in a community repository (e.g. GitHub). See the Nature Portfolio guidelines for submitting code & software for further information.

Data

Policy information about availability of data

All manuscripts must include a data availability statement. This statement should provide the following information, where applicable:

- Accession codes, unique identifiers, or web links for publicly available datasets
- A description of any restrictions on data availability
- For clinical datasets or third party data, please ensure that the statement adheres to our policy

Data is available in the source data file for data associated with the main figures. Data associated with the supplementary figures is available in the supplementary data, except for data associated with FigS6 which are available as a separate document. All additional raw data are available upon request to the author. Plasmid sequences are available on NCBI. Biological materials are available in AddGene, ABRC or upon request.

Human research participants

Policy information about studies involving human research participants and Sex and Gender in Research.

Reporting on sex and gender	n/a
Population characteristics	n/a
Recruitment	n/a
Ethics oversight	n/a

Note that full information on the approval of the study protocol must also be provided in the manuscript.

Field-specific reporting

Please select the one below that is the best fit for your research. If you are not sure, read the appropriate sections before making your selection.

☒ Life sciences ☐ Behavioural & social sciences ☐ Ecological, evolutionary & environmental sciences

For a reference copy of the document with all sections, see nature.com/documents/nr-reporting-summary-flat.pdf

Life sciences study design

All studies must disclose on these points even when the disclosure is negative.

Sample size	No sample size calculations were performed. For Arabidopsis experiments, with the data being qualitative, the sample size was determined empirically with a minimum of 10 seedlings per characterized line. For Tobacco experiments, data come from three experiments on different days, in each experiment, with three leaves on separate plants with three punches per leaf.
Data exclusions	The only excluded data were from plants/seedlings which did not grow properly, or for tobacco experiments with unsuccessful injection (no RFP expression with the positive control).
Replication	All findings can be reproduced. To verify reproducibility, for Arabidopsis experiments, a minimum of 14 seedlings were characterized. For T1 lines, seedlings were characterized on different days. We show the variability in the plant lines that we characterized by showing the characterization data of multiple T1, T2, (and for some T3, T4) lines per construct. For Tobacco experiments, data come from three experiments on different days, in each experiment, three leaves on separate plants with three punches per leaf
Randomization	For Arabidopsis experiments, we didn't have experimental groups. We characterized all seedlings planted. Genotypes were randomized during growth. For Tobacco injections, leaves and plants were randomly selected for injection of a specific construct. For each experiment, negative and positive controls were run in parallel.
Blinding	For Tobacco experiments, measurements were performed by a plate reader so blinding for data collection was not relevant as it was done by a machine following a set protocol. Data analysis was performed via a python script. For Arabidopsis experiments, all data were collected via microscopy, and microscopy was performed without knowing the construct being characterized. For image processing, an ImageJ script was used to make the analysis uniform for all images.

Reporting for specific materials, systems and methods

We require information from authors about some types of materials, experimental systems and methods used in many studies. Here, indicate whether each material, system or method listed is relevant to your study. If you are not sure if a list item applies to your research, read the appropriate section before selecting a response.

Materials & experimental systems

n/a	Involved in the study
☒	☐ Antibodies
☒	☐ Eukaryotic cell lines
☒	☐ Palaeontology and archaeology
☒	☐ Animals and other organisms
☒	☐ Clinical data
☒	☐ Dual use research of concern

Methods

n/a	Involved in the study
☒	☐ ChIP-seq
☒	☐ Flow cytometry
☒	☐ MRI-based neuroimaging
